# Screening a Compound Library to Identify Additives That Boost Cytochrome P450 Enzyme Function in Vascularised Liver Spheres

**DOI:** 10.3390/cells13181594

**Published:** 2024-09-22

**Authors:** Baltasar Lucendo-Villarin, Yu Wang, Sunil K. Mallanna, Erin A. Kimbrel, David C. Hay

**Affiliations:** 1 Centre for Regenerative Medicine, Institute for Regeneration and Repair, The University of Edinburgh, Edinburgh BioQuarter, Edinburgh EH16 4UU, UK; balta.lucendo@gmail.com (B.L.-V.); wangyu06sea@gmail.com (Y.W.); 2 Astellas Institute for Regenerative Medicine, 9 Technology Drive, Westborough, MA 01581, USA; sunil.mallanna@satellite.bio; 3 Satellite Biosciences, 580 Pleasant Street, Watertown, MA 02472, USA

**Keywords:** pluripotent stem cell, liver, hepatocyte function, screening, small molecules, regenerative medicine

## Abstract

To accurately study human organ function and disease ‘in the dish’, it is necessary to develop reliable cell-based models that closely track human physiology. Our interest lay with the liver, which is the largest solid organ in the body. The liver is a multifunctional and highly regenerative organ; however, severe liver damage can have dire consequences for human health. A common cause of liver damage is adverse reactions to prescription drugs. Therefore, the development of predictive liver models that capture human drug metabolism patterns is required to optimise the drug development process. In our study, we aimed to identify compounds that could improve the metabolic function of stem cell-derived liver tissue. Therefore, we screened a compound library to identify additives that improved the maturity of *in vitro*-engineered human tissue, with the rationale that by taking such an approach, we would be able to fine-tune neonatal and adult cytochrome P450 metabolic function in stem cell-derived liver tissue.

## 1. Introduction

To model human health and disease ‘in the dish’, it is necessary to develop cell-based systems that accurately model human physiology [1]. The liver is a multifunctional and highly regenerative organ. However, in both the acute and chronic settings, liver damage has dire consequences for human health. A common cause of liver damage is adverse reactions to prescription drugs, which can lead to drug-induced liver injury [2]. Therefore, the development of high-fidelity human liver tissue models, which accurately predict human drug metabolism, is required to highlight any potential off-target drug effects in humans. Key enzymes in human drug development are the cytochrome P450s (CYP P450s). Our ability to better understand their activity and interaction in drug metabolism is key. This will not only lead to safer drug development, but also could significantly reduce the cost and timeline of this process.

Historically, to model the function of the liver, researchers centred on the major functional cell type of the liver, the hepatocyte [3]. Although primary human hepatocytes (PHHs) are regarded as the current gold standard model to study human drug metabolism and toxicity, they display instability and loss of phenotype in culture and are a scarce and expensive resource due to isolation from transplant reject organs [4]. To bypass these issues, many basic and industrial studies have used transformed cell lines to scale up liver-like tissue for biomedical application. However, it is well accepted that those models display compromised levels of hepatocyte function [5]. Therefore, researchers have developed 3D liver models, which are more stable phenotypically; however, they remain variable in nature within and between experiments [6].

For models to have significant impact, they must address the issues with the phenotype and variation, as well as be capable of production from genetically defined origins, which are renewable in nature. Such a resource would provide the user with quality-assured and stable human liver tissue for application. This is of particular importance for human drug development. For this reason, we opted to use induced pluripotent stem cells (iPSCs) to build renewable and donor-free source of human liver tissue. Specifically, 2D hepatocyte-like cells (HLCs) [7] and 3D liver spheres [4], containing hepatocytes and endothelial cells, were generated in vitro. They were used to screen a compound library with the aim of improving stem cell-derived HLCs and vascularized liver sphere maturity and metabolic function [7]. 

In summary, we used pluripotent stem cell-derived liver tissue to screen a small-molecule library. The goal was to identify compounds that stimulated hepatocyte maturation and CYP450 metabolic function. We reasoned that by taking such an approach, we could fine-tune stem cell-derived liver tissue performance, which could be important for human drug development in the future.

## 2. Materials and Methods

### 2.1. Cell Culture and Differentiation

The human-induced pluripotent stem cell line JHU106i (WiCell Research Institute, Madison, WC, USA) was cultured on Laminin 521 (BioLamina, Sundbyberg, Sweden)-coated plates in serum-free mTeSR1™ (STEMCELL Technologies, Vancouver, Canada) in an antibiotic-free medium and in a humidified 37 °C, 5% CO_2_ incubator as previously described [8]. 

#### 2.1.1. Hepatic Differentiation

Induced pluripotent stem cells (iPSCs) were differentiated towards liver progenitor cells as previously described [8]. Briefly, following cell dissociation using Gentle Cell Dissociation Reagent (STEMCELL technologies, Vancouver, Canada), iPSC cells dissociated into single cells were plated onto pre-coated wells with Laminin 521 (BioLamina, Sundbyberg, Sweden) in mTeSR1™ supplemented with 10 µM Y-27632 (Biotech, Minneapolis, MN, USA) at a density of 40,000 cells·cm^−2^. Differentiation was initiated 24 h post seeding by replacing the stem cell medium with endoderm differentiation medium supplemented with 100 ng·mL^−1^ Activin A (Biotechne, Minneapolis, MN, USA) and 50 ng·mL^−1^ Wnt3a (Biotechne, Minneapolis, MN, USA) for 3 days with medium changes every 24 h. On day 3, cell culture mediium was switched to hepatic progenitor differentiation medium, and this was renewed every 48 h for a further 5 days. On day 9, differentiating cells were cultured for 24 h in hepatocyte maturation medium comprised of Hepato-ZYME (Life Technologies, Carlsbad, CA, USA) supplemented with 10 ng·mL^−1^ hepatocyte growth factor (PeproTech, Drive Cranbury, NJ, USA) and 20 ng·mL^−1^ oncostatin M (PeproTech, Drive Cranbury, NJ, USA).

#### 2.1.2. Endothelial Cell Differentiation

Endothelial differentiation was performed using an adapted protocol previously described [9]. Briefly, following cell dissociation using Gentle Cell Dissociation Reagent (STEMCELL technologies, Vancouver, Canada), iPSCs dissociated into single cells were plated onto pre-coated wells with Laminin 521 (BioLamina, Sundbyberg, Sweden) in mTeSR1™ supplemented with 10 µM Y-27632 (Biotechne, Minneapolis, MN, USA) at a density of 25,000 cells·cm^−2^. After 24 h, the media was replaced with mesoderm priming medium supplemented with 10 µM CHIR99021 (Tocris, Bristol, UK) and 25 ng·mL^−1^ BMP4 (Biotechne, Minneapolis, MN, USA) for 72 h. Following this, the priming medium was replaced by endothelial induction medium consisting of StemPro-34 SFM medium (Life Technologies, Carlsbad, CA, USA) supplemented with 200 ng·mL^−1^ vascular endothelial growth factor (Biotechne, Minneapolis, MN, USA) and 2 µM forskolin (Sigma-Aldrich, San Luis. MO, USA). The induction medium was renewed every 24 h for a further 2 days.

### 2.2. Automated Production of Liver Spheres 

The automated aggregation of vascularised liver spheres was performed as previously described [7]. Briefly, following cell detachment using TrypLE express (ThermoFisher, Carlsbad, CA, USA), hepatic progenitor-like cells (HBs) were filtered using a 30 µM cell strainer to obtain a single-cell suspension, centrifugated and resuspended in liver sphere medium supplemented with 10 µM Y-27632 (Biotechne, Minneapolis, MN, USA), 10 ng·mL^−1^ epithelial growth factor (Biotechne, Minneapolis, MN, USA), 10 ng·mL^−1^ fibroblast growth factor (PeproTech, Drive Cranbury, NJ, USA), 10 ng·mL^−1^ hepatocyte growth factor (PeproTech, Drive Cranbury, NJ, USA), 20 ng·mL^−1^ oncostatin M (PeproTech, Drive Cranbury, NJ, USA), and 50 ng·mL^−1^ vascular endothelial growth factor (Biotechne, Minneapolis, MN, USA) at a density of 10.95 × 10^6^ live cells·mL^−1^. For endothelial enrichment, magnetic-associated cell sorting (MACS) technology using CD144 MicroBeads (Miltenyi Biotec, Bisley, Surrey, UK) was employed, and the endothelial-like cells (ELCs) were resuspended in growth factor- and Y-27632-supplemented liver sphere medium at a density of 7.13 × 10^6^ live cells·mL^−1^. 

Following this, a cell mixture containing 1 volume of the hepatic progenitor cell solution and 0.5 volumes of the endothelial cell solution was prepared to a final cell ratio of 10:3 (hepatic progenitor/endothelial cells). The multidrop system (ThermoFisher 5840300, Carlsbad, CA, USA) and Viaflo 96 head pipette system (Integra 6001, Integra Biosciences, Thatcham, Berkshire, UK) technologies were combined to seed the cell solution into a 96-well hydrogel array containing 73 microwells (500 µM) per micromold (Gri3D, SUNbiosciences, Lausanne, Switzerland). 2 h post-aggregation, 200 µL of liver sphere medium was added into the feeding chamber and media changes were performed every 48 h. 

### 2.3. Compound Exposure

**Figure 1:**
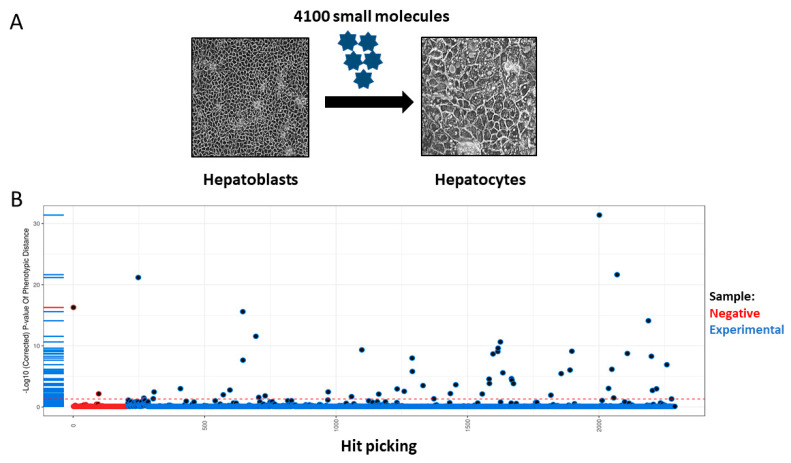
(**A**) Summary of compound screening and hit picking. A library of 4100 compounds were screened using human pluripotent stem cell-derived hepatocyte-like cells (HLCs). (**B**) During the 8 days of hepatocellular differentiation, the cells were exposed to 1 µM of each compound. Supernatants were harvested at the end of differentiation and AFP secretion was used to determine cell maturity [7]. Hit compounds were selected based on their ability to reduce AFP secretion at day 18 in HLCs.

Using our developed 2D hepatocyte-like cell model derived from pluripotent stem cells [10], we screened a library of 4100 commercially available, non-overlapping compounds containing various FDA- and EMA-approved drugs, natural products, and other bioactive compounds that target kinases, ion channels, nuclear receptors, epigenetic regulators, G-protein-coupled receptors, and those reported to impact stem cell differentiation, proliferation, reprogramming, and signalling (sourced from a combination of Microsource (Gaylordsville, CT, USA) and Tocris (Bristol, UK) collections). Following our initial screen, we identified compounds that promoted hepatocyte-like cell maturity with a decrease in alpha-fetoprotein (AFP) secretion detected (summarised in Figure 1 and assay performed as before [7]). We selected the top ten compound hits from our 2D analysis to study their effects in 3D-engineered vascularized liver spheres, as this is likely to be more representative of human liver tissue. 3D vascularized liver spheres produced from induced pluripotent stem cells were exposed to compounds resuspended in dimethyl sulfoxide (DMSO–Sigma Aldrich, San Luis. MO, USA) and diluted to 1:1000 in liver spheroid medium. The concentration range was from 0.1 µM to 10 µM. Liver spheres at day 14 were exposed to each compound for 72 h. Medium supplemented with 0.1% DMSO was used as the vehicle control. 

### 2.4. Cell Health in Response to Hit Compounds

Post-exposure, the compound effect on cell health was determined by evaluating cell viability and apoptosis using RealTime-Glo MT Cell Viability Assay (Promega, Madison, WI, USA) and Caspase 3/7 Assay System (Promega, Madison, WI, USA), respectively. In total, 100 μL of the CellTiter-Glo luciferase substrate or Caspase 3/7 assay was mixed with 100 μL of culture medium in the plate containing the liver spheres. Four individual micromolds were employed per assay and each well was read in duplicate using a multiplex plate reader (GloMax Explorer, Promega, Madison, WI, USA) and normalised and compared to the vehicle control (DMSO). Results represent the mean ± SD.

### 2.5. Cytochrome P450 Activity in Response to Hit Compounds

CYP3A4 and CYP1A2 enzyme activities are key for adult liver function and prescription drug metabolism. Both activities were measured in liver spheres using the P450-Glo™ assay (Promega, Madison, WI, USA) using luciferin-PFBE substrate (Promega, Madison, WI, USA) or luciferin-ME substrate (Promega, Madison, WI, USA), respectively. Following the manufacturer’s instructions, CYP P450 substrates were diluted in liver sphere medium. Activity was measured 24 h post-addition. Four individual micromolds were employed per assay and each well was read in triplicate to ensure quality, using a multiplex plate reader (GloMax Explorer, Promega, Madison, WI, USA). Relative light units were normalised to per milligram of protein and compared to the vehicle control.

### 2.6. Statistics

One-way ANOVA statistical analysis was performed in Figures 4 and 5 using GraphPad Prism software, version 9.5.1 (733). * *p* < 0.05, ** *p* < 0.01, *** *p* < 0.001, and **** *p* < 0.0001 versus vehicle control. 

## 3. Results

We screened a compound library of 4100 commercially available small molecules with the goal to improve stem cell-derived HLC maturity (Figure 1). Human PSCs were differentiated to hepatic progenitors and exposed to each compound (1 µM). The compounds were reconstituted in cell culture medium and this was replaced every 48 h over an 8-day time course. In order to identify improvements in hepatocellular maturity, we measured the secretion of AFP in HLCs, a fetal marker. Stem cell-derived HLCs that displayed reduced AFP secretion, were presumed to be more mature. From those compound hits, we selected the top ten to study in further detail using our 3D semi-automated liver sphere platform (Table 1). Liver spheres were composed of hepatic progenitors and endothelial cells and self-assembled into 3D structures in microwells. Fourteen days post liver sphere formation, they were exposed to hit compounds for 72 h with caspase activity, cell viability, and cytochrome P450 metabolic activities recorded.

**Figure 2:**
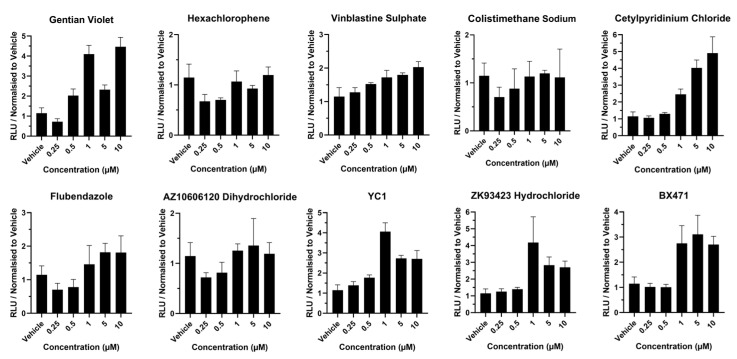
Caspase 3/7 activity in liver spheres following 72 h compound exposure. Three-dimensional vascularized liver spheres were produced from pluripotent stem cells and exposed to compounds resuspended in dimethyl sulfoxide (DMSO) and diluted to 1:1000 in liver spheroid medium. Concentration range was from 0.25 µM to 10 µM. Liver spheres at day 14 were exposed to each compound for 72 h. Medium supplemented with 0.1% DMSO was used as vehicle control. Data represent mean ± SD (*n* = 4).

Following exposure to gentian violet, caspase activity was increased 2-fold at 0.5 µM and 5 µM concentrations and 3.5-fold between 1 and 10 µM concentrations (Figure 2), whereas exposure to hexachlorophene, vinblastine sulphate, colistimethate sodium, flubendazole, and AZ10606120 dihydrochloride did not induce large changes in caspase activity across the concentration range (Figure 2). Following exposure to cetylpyridinium chloride, YC1 and BX471 caspase activity increased (>2-fold) at concentrations of 1 µM and above (Figure 2).

**Figure 3:**
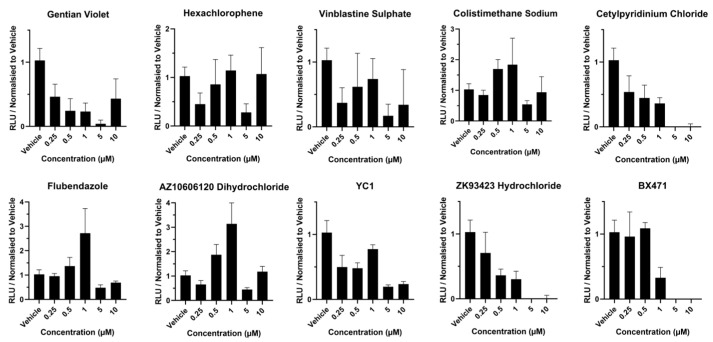
ATP levels in liver spheres following 72 h compound exposure. Three-dimensional vascularized liver spheres were produced from pluripotent stem cells and exposed to compounds resuspended in dimethyl sulfoxide (DMSO) and diluted to 1:1000 in liver spheroid medium. Concentration range was from 0.25 µM to 10 µM. Liver spheres at day 14 were exposed to each compound for 72 h. Medium supplemented with 0.1% DMSO was used as vehicle control. Data represent mean ± SD (*n* = 4).

Next, we studied the effects of compound exposure on liver sphere viability. Following exposure to gentian violet, hexachlorophene, and vinblastine sulphate at 0.25 µM, we observed >50% of ATP depletion (Figure 3), whereas following exposure to cetylpyridinium chloride at 0.50 µM and above, we observed >50% ATP depletion (Figure 3). Exposure to colistimethate sodium, flubendazole, and AZ10606120 dihydrochloride at 5 µM resulted in >50% depletion (Figure 3), whereas exposure to YC1 at 0.25 µM and 0.5 µM resulted in significant levels of ATP depletion (>50%) with a further reduction following exposure to 5 µM and 10 µM concentrations (Figure 3). Following exposure to ZK93423 hydrochloride at 0.5 µM and above, we observed significant levels of ATP depletion (>50%), with liver spheres displaying no detectable levels of ATP at 5 and 10 µM exposure (Figure 3). Following exposure to BX471 at 1 µM, we observed significant levels of ATP depletion (>50%) (Figure 3).

**Figure 4:**
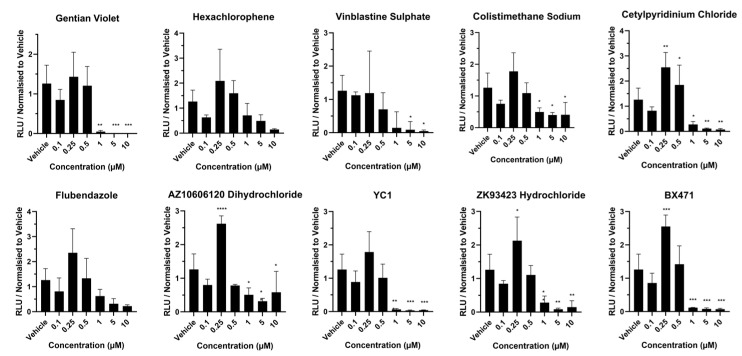
CYP1A2 activity in liver spheres following 72 h compound exposure. Three-dimensional vascularized liver spheres were produced from pluripotent stem cells and exposed to compounds resuspended in dimethyl sulfoxide (DMSO) and diluted to 1:1000 in liver spheroid medium. Concentration range was from 0.1 µM to 10 µM. Liver spheres at day 14 were exposed to each compound for 72 h. Medium supplemented with 0.1% DMSO was used as vehicle control. Data represent mean ± SD (*n* = 4). * *p* < 0.05, ** *p* < 0.01 and *** *p* < 0.001, versus vehicle control.

**Figure 5:**
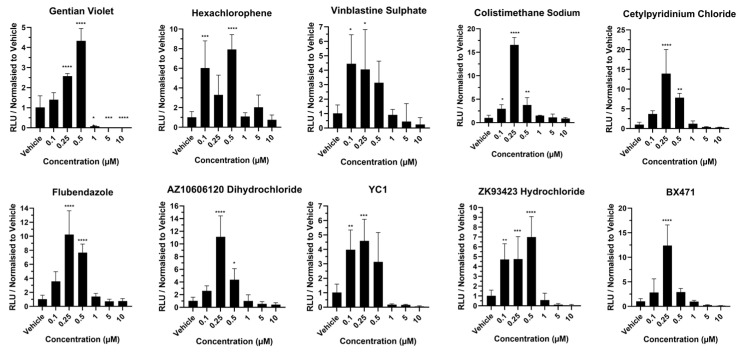
CYP3A4 activity in liver spheres following 72 h compound exposure. Three-dimensional vascularized liver spheres were produced from pluripotent stem cells and exposed to compounds resuspended in dimethyl sulfoxide (DMSO) and diluted to 1:1000 in liver spheroid medium. Concentration range was from 0.1 µM to 10 µM. Liver spheres at day 14 were exposed to each compound for 72 h. Medium supplemented with 0.1% DMSO was used as vehicle control. Data represent mean ± SD (*n* = 4). * *p* < 0.05, ** *p* < 0.01, *** *p* < 0.001, and **** *p* < 0.0001 versus vehicle control.

The goal of our study was to identify compounds that could improve liver cell metabolic function with a focus on cytochrome P450 function relevant for prescription drug metabolism. Cytochrome P450 3A4 and 1A2 activities were studied. Following exposure to gentian violet, CYP1A2 activity did not increase over the concentration range, and decreased at >1 µM (Figure 4), whereas CYP3A4 function increased ~4-fold at 0.5 µM, but was at the baseline at concentrations above this (Figure 5). Following exposure to hexachlorophene, CYP1A2 activity did not increase over the concentration range, and decreased at >5 µM (Figure 4). In contrast, CYP3A4 activity was increased between 6- and 7-fold at 0.10 and 0.5 µM (Figure 5). In response to vinblastine sulphate, CYP1A2 activity did not increase over the concentration range, and decreased to the baseline at concentrations above 1 µM (Figure 4), whereas CYP3A4 activity was increased between 3- and 4-fold between 0.10 and 0.5 µM concentration (Figure 5). CYP1A2 activity did not increase over the colistimethate sodium concentration range (Figure 4). However, CYP3A4 activity increased by 16-fold at 0.25 µM, but at concentrations above this, metabolic activity was at the baseline (Figure 5). Notably, post-exposure to cetylpyridinium chloride, CYP1A2 activity was increased ~2-fold at 0.25 and 0.5 µM but reached the baseline at concentrations above this (Figure 4). CYP3A4 activity showed a 16- and 8-fold increase at 0.25 and 0.5 µM, respectively, but at concentrations >1 µM, the metabolic activity approached the baseline (Figure 5). Similarly, exposure to flubendazole resulted in 2-fold-increased CYP1A2 activity at 0.25 µM concentration but decreased to the baseline above 1 µM (Figure 4), whereas CYP3A4 activity was increased between 8- and 10-fold at 0.25 and 0.5 µM concentration (Figure 5). Post AZ10606120 dihydrochloride exposure, CYP1A2 activity was increased ~2-fold at 0.25 µM concentration but was at the baseline at concentrations above this (Figure 4). CYP3A4 activity increased by 10- and 4-fold at 0.25 and 0.5 µM, respectively, but was minimal at concentrations >1 µM (Figure 5). Following exposure to YC1, CYP1A2 activity increased ~1.5-fold at 0.25 µM, but was at the baseline at concentrations above this (Figure 4), whereas CYP3A4 activity was increased between 3- and 4-fold between 0.10 and 0.5 µM but decreased to the baseline at concentrations above 1 µM (Figure 5). Exposure to ZK93423 hydrochloride led to a 2-fold increase in CYP1A2 activity at 0.25 µM concentration, but was at the baseline above this concentration (Figure 4), whereas CYP3A4 activity was increased between 5- and 7-fold between 0.10 and 0.5 µM but decreased to the baseline above >1 µM (Figure 5). Exposure to BX471 led to a 2.5-fold increase in CYP1A2 activity at 0.25 µM, but was at the baseline at concentrations above this (Figure 4), whereas CYP3A4 activity was increased 12-fold at 0.25 µM, but decreased to the baseline at concentrations greater than 1 µM (Figure 5). Statistical significance is detailed on the graphs (Figure 4 and Figure 5) and these data are summarised in Table 2, highlighting colistimethate sodium, flubendazole, and BX471 as the most promising compounds to improve hepatocellular metabolic activity when used at non-toxic concentrations.

## 4. Discussion

The purpose of this study was to identify compounds that improved the metabolic activity of stem cell-derived liver spheres at non-toxic concentrations. This is an important consideration as current models using human liver tissue suffer from rapid loss of function and viability during ex vivo culture [11]. Unstable phenotype is also observed in stem cell-based liver systems, in particular 2D hepatocyte-like models. Additionally, transformed cell lines used in experimentation display substandard levels of human enzyme function [5]. These inadequacies are problematic as they may compromise the efficiency and safety of the drug development process. As a result, the average cost of human drug development for the clinic is estimated to be between USD 1 and 2 billion [12]. In an attempt to address this, we used stem cell-based models of human liver tissue to screen a small-molecule library. Our goal was to use these molecules to improve cell maturity and fine-tune metabolic function, in particular cytochrome P450s (CYP P450), which orchestrate prescription drug metabolism. 

Liver spheres were exposed to the top ten compounds identified from a previous screen of 4100 compounds (Figure 1, Table 1). In those studies, compounds were selected on the basis of reduced HLC AFP secretion (summarised in Figure 1). Seven of the compounds employed demonstrated poor characteristics, which included increased caspase activity, reduced cell viability, and significant changes in CYP P450 function. Those compounds were gentian violet, an antifungal used to treat infections inside the mouth and of the skin [13]; hexachlorophene, a topical antibacterial cleanser [14]; vinblastine sulphate, an antineoplastic agent used to treat certain kinds of cancer [15]; cetylpyridinium chloride, an antibacterial agent used to treat gingivitis [16]; AZ 10606120 dihydrochloride, an antagonist of P2X7 activation known to significantly reduce the fibrosis of the liver [17]; YC1, a nitric oxide-independent activator of soluble guanylyl cyclase, a therapeutic target in cardiopulmonary disease [18]; and finally ZK 93423 hydrochloride, a GABAA agonist, anticonvulsant, and muscle relaxant [19].

In contrast, three compounds demonstrated favourable traits, when used at non-toxic concentrations. Those compounds were colistimethate sodium, a broad-spectrum antibiotic [20]; flubendazole, which has been repurposed to inhibit cancer cell growth [21]; and BX471, a CCR1 antagonist, which has shown promise in modulating proinflammatory and autoimmune diseases [22]. Following exposure, stem cell-derived liver spheres exhibited low levels of caspase activity and good levels of viability, in concert with significantly increased levels of CYP 3A4 function. This is important as CYP 3A4 is estimated to metabolise approximately 50% of clinically approved drugs. The pattern was similar for CYP1A2 function, but only reached statistical significance with BX471. Although stem cell-derived liver spheres provide a useful and renewable model, they do suffer from limitations, such as incomplete function and fetal protein secretion when compared to PHHs. Therefore, the ability to reduce fetal protein secretion and improve drug metabolism marks a significant step forward using PSC-derived liver tissue. Moving beyond these studies, it will be important to test the efficacy of these compounds, in isolation and in combination, on stem cell-derived material with a greater number of replicates, as well as testing on other cell-based systems, including PHHs and liver cell lines.

In conclusion, we screened a compound library to identify the promising candidates that could regulate human drug metabolising enzyme function. From an initial 4100 compounds, we tested the top ten hits on stem cell-derived vascularised liver spheres. From the ten, we identified three promising candidates that reduced fetal protein secretion and fine-tuned CYP P450 function, in particular CYP3A4. We believe that these compounds could be important additives to cell culture medium to improve the liver cell phenotype for human drug metabolism and safety studies.

## Figures and Tables

**Table 1:** Compound names and supplier details.

Compound Name	Vendor	Catalogue Number
Gentian Violet	Microsource Discovery	01500315
Hexachlorophene	Microsource Discovery	01500328
Vinblastine Sulphate	Microsource Discovery	01500611
Colistimethane Sodium	Microsource Discovery	01500206
Cetylpyridinium Chloride	Microsource Discovery	01500169
Flubendazole	Microsource Discovery	01505043
AZ10606120 Dihydrochloride	Tocris	3323
YC1	Tocris	4307
ZK93423 Hydrochloride	Tocris	1994
BX471	Tocris	3496

**Table 2:** Concentration of compound and effects on cell viability, caspase activation, and CYP1A2 and CYP3A4 activity.

Compound	Viability Reduction at 50%	Peak Caspase Activity	Peak CYP1A2 Activity	Peak CYP3A4 Activity
Gentian Violet	0.25 µM	0.50 µM	0.25 µM	0.50 µM
Hexachlorophene	0.25 µM	>10 µM	0.25 µM	0.50 µM
Vinblastine Sulphate	0.25 µM	>10 µM	0.25 µM	0.10 µM
Colistimethane Sodium	5 µM	>10 µM	0.25 µM	0.25 µM
Cetylpyridinium Chloride	0.25 µM	>5 µM	0.25 µM	0.25 µM
Flubendazole	5 µM	5 µM	0.25 µM	0.25 µM
AZ10606120 Dihydrochloride	0.25 µM	>10 µM	0.25 µM	0.25 µM
YC1	0.25 µM	>1 µM	0.25 µM	0.25 µM
ZK93423 Hydrochloride	0.50 µM	>1 µM	0.25 µM	0.50 µM
BX471	1 µM	5 µM	0.25 µM	0.25 µM

## Data Availability

All data generated or analyzed during this study are included in this article.
